# Supplementary material for: Empirical methods for controlling false positives and estimating confidence in ChIP-Seq peaks
Source: BMC Bioinformatics. 2008 Dec 5;9:523. doi: 10.1186/1471-2105-9-523 (PMC2628906; doi:10.1186/1471-2105-9-523)
Supplement: Additional File 1 — A variety of html documents from the USeq web site detailing the available applications, their best usage, output file type descriptions, command line menus, etc. [file 1471-2105-9-523-S1.zip › USeqUserGuides/JavaDoc.zip!/JavaDoc/bioroot/Archive.html]

Archive


|  |  |  |
| --- | --- | --- |
| **Overview** \| **Package** \| **Class** \| **Use** \| **Tree** \| **Deprecated** \| **Index** \| **Help** | |  |
| **PREV CLASS**   **NEXT CLASS** | **FRAMES**    **NO FRAMES**     **All Classes** |  |
| SUMMARY: NESTED \| FIELD \| CONSTR \| METHOD | DETAIL: FIELD \| CONSTR \| METHOD |  |


---


## bioroot Class Archive

```
java.lang.Object
  javax.servlet.GenericServlet
      javax.servlet.http.HttpServlet
          bioroot.Archive
```

**All Implemented Interfaces:**: java.io.Serializable, javax.servlet.Servlet, javax.servlet.ServletConfig

---

``` public class Archive extends javax.servlet.http.HttpServlet ```

**Author:**
:   nix
    Creates a tab delimited text file for download, a full copy of a users collection limited by user type.

**See Also:**: Serialized Form

---

**Constructor Summary**

`Archive()`


| **Method Summary** | |
| --- | --- |
| `static java.lang.String` | `buildSqlStatement(UserBean user, java.lang.String tableName)`             Creates a sql statement for super users and standard users returns null for everyone else, lab specific. |
| `static java.lang.String` | `buildTabTableRows(java.lang.String[][] columnRows)`             Build tab delimited table rows. |
| `void` | `doGet(javax.servlet.http.HttpServletRequest request, javax.servlet.http.HttpServletResponse response)` |
| `void` | `doPost(javax.servlet.http.HttpServletRequest request, javax.servlet.http.HttpServletResponse response)` |
| `static java.util.ArrayList` | `fetchFiles(PlasmidBean[] plasmids, StrainBean[] strains, AntibodyBean[] antibodies, java.lang.String filePath, DBUtil bioRoot)`             Fetches any plasmid or strain files. |
| `static java.lang.String` | `fetchTabbedTableColumnRow(Feature[] features)`             Returns a table header row using the Preferece obj |
| `static AntibodyBean[]` | `makeAntibodyBeans(DBUtil bioRoot, UserBean userBean)`             Makes an array of all visibile AntibodyBeans |
| `static OligoBean[]` | `makeOligoBeans(DBUtil bioRoot, UserBean userBean)`             Makes an array of all visibile OligoBeans |
| `static PlasmidBean[]` | `makePlasmidBeans(DBUtil bioRoot, UserBean userBean)`             Makes an array of all visibile PlasmidBeans |
| `static StrainBean[]` | `makeStrainBeans(DBUtil bioRoot, UserBean userBean)`             Makes an array of all visibile StrainBeans |

**Methods inherited from class javax.servlet.http.HttpServlet**

`service`

**Methods inherited from class javax.servlet.GenericServlet**

`destroy, getInitParameter, getInitParameterNames, getServletConfig, getServletContext, getServletInfo, getServletName, init, init, log, log`

**Methods inherited from class java.lang.Object**

`equals, getClass, hashCode, notify, notifyAll, toString, wait, wait, wait`

**Constructor Detail**

### Archive

```
public Archive()
```


**Method Detail**

### doGet

```
public void doGet(javax.servlet.http.HttpServletRequest request,
                  javax.servlet.http.HttpServletResponse response)
           throws javax.servlet.ServletException,
                  java.io.IOException
```

:   **Overrides:**: `doGet` in class `javax.servlet.http.HttpServlet`
:   **Throws:**: `javax.servlet.ServletException`: `java.io.IOException`

---


### doPost

```
public void doPost(javax.servlet.http.HttpServletRequest request,
                   javax.servlet.http.HttpServletResponse response)
            throws javax.servlet.ServletException,
                   java.io.IOException
```

:   **Overrides:**: `doPost` in class `javax.servlet.http.HttpServlet`
:   **Throws:**: `javax.servlet.ServletException`: `java.io.IOException`

---


### fetchFiles

```
public static java.util.ArrayList fetchFiles(PlasmidBean[] plasmids,
                                             StrainBean[] strains,
                                             AntibodyBean[] antibodies,
                                             java.lang.String filePath,
                                             DBUtil bioRoot)
```

:   Fetches any plasmid or strain files.

---


### makeOligoBeans

```
public static OligoBean[] makeOligoBeans(DBUtil bioRoot,
                                         UserBean userBean)
```

:   Makes an array of all visibile OligoBeans

---


### makePlasmidBeans

```
public static PlasmidBean[] makePlasmidBeans(DBUtil bioRoot,
                                             UserBean userBean)
```

:   Makes an array of all visibile PlasmidBeans

---


### makeAntibodyBeans

```
public static AntibodyBean[] makeAntibodyBeans(DBUtil bioRoot,
                                               UserBean userBean)
```

:   Makes an array of all visibile AntibodyBeans

---


### makeStrainBeans

```
public static StrainBean[] makeStrainBeans(DBUtil bioRoot,
                                           UserBean userBean)
```

:   Makes an array of all visibile StrainBeans

---


### fetchTabbedTableColumnRow

```
public static java.lang.String fetchTabbedTableColumnRow(Feature[] features)
```

:   Returns a table header row using the Preferece obj

---


### buildTabTableRows

```
public static java.lang.String buildTabTableRows(java.lang.String[][] columnRows)
```

:   Build tab delimited table rows.

---


### buildSqlStatement

```
public static java.lang.String buildSqlStatement(UserBean user,
                                                 java.lang.String tableName)
```

:   Creates a sql statement for super users and standard users returns null for everyone else, lab specific.


---


|  |  |  |
| --- | --- | --- |
| **Overview** \| **Package** \| **Class** \| **Use** \| **Tree** \| **Deprecated** \| **Index** \| **Help** | |  |
| **PREV CLASS**   **NEXT CLASS** | **FRAMES**    **NO FRAMES**     **All Classes** |  |
| SUMMARY: NESTED \| FIELD \| CONSTR \| METHOD | DETAIL: FIELD \| CONSTR \| METHOD |  |


---
